# Sensing, assessing, and augmenting threat detection: behavioral, neuroimaging, and brain stimulation evidence for the critical role of attention

**DOI:** 10.3389/fnhum.2013.00273

**Published:** 2013-06-12

**Authors:** Raja Parasuraman, Scott Galster

**Affiliations:** ^1^Center of Excellence in Neuroergonomics, Technology and Cognition, George Mason UniversityFairfax, VA, USA; ^2^Applied Neuroscience Branch, Air Force Research LaboratoryDayton, OH, USA

**Keywords:** action understanding, attention, biological motion, brain stimulation, human performance augmentation, neuroimaging, security, threat detection

## Abstract

Rapidly identifying the potentially threatening movements of other people and objects—biological motion perception and action understanding—is critical to maintaining security in many civilian and military settings. A key approach to improving threat detection in these environments is to sense when less than ideal conditions exist for the human observer, assess that condition relative to an expected standard, and if necessary use tools to augment human performance. Action perception is typically viewed as a relatively “primitive,” automatic function immune to top-down effects. However, recent research shows that attention is a top-down factor that has a critical influence on the identification of threat-related targets. In this paper we show that detection of motion-based threats is attention sensitive when surveillance images are obscured by other movements, when they are visually degraded, when other stimuli or tasks compete for attention, or when low-probability threats must be watched for over long periods of time—all features typical of operational security settings. Neuroimaging studies reveal that action understanding recruits a distributed network of brain regions, including the superior temporal cortex, intraparietal cortex, and inferior frontal cortex. Within this network, attention modulates activation of the superior temporal sulcus (STS) and middle temporal gyrus. The dorsal frontoparietal network may provide the source of attention-modulation signals to action representation areas. Stimulation of this attention network should therefore enhance threat detection. We show that transcranial Direct Current Stimulation (tDCS) at 2 mA accelerates perceptual learning of participants performing a challenging threat-detection task. Together, cognitive, neuroimaging, and brain stimulation studies provide converging evidence for the critical role of attention in the detection and understanding of threat-related intentional actions.

## Introduction

Rapidly detecting and identifying the movements and actions of other people—biological motion perception—is an important function of many civilian and military operational settings involving surveillance and other security related tasks. For example, cameras mounted in prisons (Tickner and Poulton, 1975) and other sensitive locations (Stedmon et al., 2011), or on unmanned air (Cummings et al., 2007) and ground vehicles (Chen and Barnes, 2012), are increasingly used to provide video or infrared images to remotely located operators. Surveillance images typically show people or vehicles in motion and engaged in various activities. Such information can be used to identify individuals who pose potential threats or to determine the potential for danger in gatherings of large groups of people. The images are examined for possible threats by skilled human observers (Blake and Shiffrar, 2007), by automated systems (Cohen et al., 2008), or by a combination of the two.

Biological motion perception has typically been investigated in psychophysical studies using simple point-light “stick-figure” movements of the type pioneered by Johansson (1973). More recent studies have examined more complex, naturalistic scenes of people moving or handling objects (Blake and Shiffrar, 2007; Ortigue et al., 2009; Parasuraman et al., 2009; Grafton and Tipper, 2012; Thompson and Parasuraman, 2012). Identifying the mechanisms and neural bases of action observation when people view naturalistic scenes can advance both the theory and practice in threat detection. More broadly, understanding the mechanisms of threat detection can contribute to scientific approaches to security based on human factors/ergonomics (Nickerson, 2010), neuroscience (National Research Council, 2008), and the intersection of these two fields, neuroergonomics (Parasuraman and Rizzo, 2008; Parasuraman, 2011; Parasuraman et al., 2012).

A key approach to improving threat detection is to *sense* when less than ideal conditions exist for the human operator in a particular security environment. The next step is to *assess* the threat detection performance of the human operator with respect to a standard baseline of required capability. Subsequently, and if necessary, methods can be implemented to *augment* the human operator in case the standard is not met. In this paper we first describe this Sense—Assess—Augment framework in the context of security research and practice in the military. We then examine the perceptual and cognitive mechanisms of biological motion-based threat detection, focusing on the critical role of attention. Neuroimaging studies that point to the influence of attention are also discussed. Finally, given that humans have limited attentional capabilities, we discuss how non-invasive brain stimulation can be used to enhance threat detection and mitigate operator performance decrements.

### Sense-assess-augment framework

A number of agencies in the military are planning how best to match personnel and emerging advanced technologies that are being rapidly implemented for use in both the civilian and defense sectors. For example, in 2010, the Chief Scientist of the US Air Force released a report outlining the science and technology needs in the 2010–2030 time frame (Dahm, 2010). A key conclusion of that report was that natural human capacities are becoming increasingly mismatched to the enormous data volumes, processing capabilities, and decision speeds that computer technologies either offer or demand. Although humans today remain more capable than machines for many tasks, particularly higher-order decision making and planning, by 2030 machine capabilities may increase to the point that human capabilities will be significantly challenged in a wide array of systems and processes. It is also the case that human operators are being overloaded *today* by data that the new technologies are able to provide at ever increasing speed. Both of these trends mean that humans and machines will need to become far more closely coupled, through improved human-machine interfaces and by direct augmentation of human performance. Focused research efforts over the next decade will permit significant practical instantiations of augmented human performance. These may come from increased use of autonomous systems, from novel human-machine interfaces to couple humans more closely and more intuitively with automated systems, or from direct augmentation of humans themselves. In this paper we focus on the last of these possibilities.

**Figure 1:**
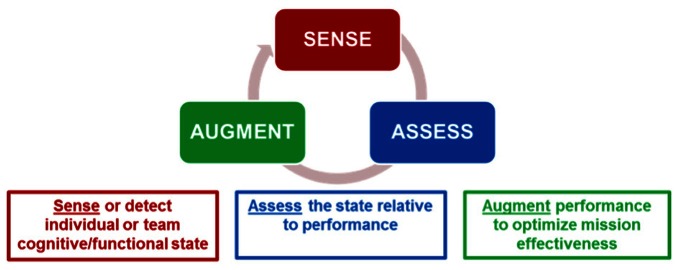
**Sense-Assess-Augment framework for optimizing threat detection performance.** From Galster and Johnson (2013).

There are two primary questions to ask when deciding how to provide human performance augmentation: *when* to provide the augmentation and *how* to provide it. The answers to these questions will likely determine if the right augmentation technique is being employed at the right time to produce the desired effects. The Sense-Assess-Augment taxonomy provides answers to these questions. Figure 1 shows a representation of this taxonomy. The objective is to sense individual and team cognitive or functional state (using behavioral or neural measures, or both), assess the state relative to performance, and if necessary augment performance to optimize mission effectiveness. This taxonomy is being applied to improve human performance by leveraging the integration of several neurocognitive sensing technologies coupled with multiple assessment approaches to provide a robust understanding of the causes of operator performance decrements (Galster and Johnson, 2013). Given a better understanding of the causes for sub-optimal performance, targeted augmentation techniques can be employed to improve individual or team performance.

Threat detection has many features in common with other tasks the Air Force undertakes to defend capabilities in its air, space, and cyberspace operations. Many of the problems associated with information overload are exacerbated in threat detection tasks due to the exponential growth in the number of pictures or full motion videos that must be processed for actionable information. The use of the Sense-Assess-Augment taxonomy allows for the identification of specific bottlenecks that may occur during the information processing of the data that is required for accurate threat detection. It also allows for the augmentation of the operator based on the characteristics of the bottleneck, for example, whether due primarily to issues with divided or sustained attention, or a lack of the ability to discriminate threats regardless of the amount of training. The correct identification of the individual's source for sub-optimal performance will drive what augmentation method should be utilized to enhance and optimize human performance.

In this paper we describe the results of a number of studies of threat detection within the Sense-Assess-Augment framework. We begin with a discussion of behavioral studies that have investigated mechanisms of biological motion perception in relation to threat detection.

## Behavioral studies

### Automaticity of biological motion perception

Sensing the movement of other biological organisms has played an important role in the survival and evolution of species. Biological threats in natural environments—such as the movements of a predator—can help an animal in the “fight or flight” response. Similarly, the predator uses this ability to sense the motion of its prey. The capacity for biological motion perception appears to be largely present at birth. Infants as young as two days old, for example, show a preference for looking at point-light displays of biological motion as opposed to random motion (Simion et al., 2008). At the other end of the lifespan, older adults, who typically exhibit an age-related decline in the efficiency of processing non-biological moving objects (Gilmore et al., 1992; Jiang et al., 1999), nevertheless have been reported to be as efficient at processing biological motion stimuli as the young (Norman et al., 2004; Billino et al., 2008).

These different lines of evidence would seem to support the notion that biological motion perception is a “primitive” and largely automatic function, although it is also possible that it is learned very early in infancy (2 days). Thornton and Vuong (2004) provided evidence for automaticity in a study using the well-known “flanker” task (Eriksen and Eriksen, 1974). Participants had to determine the direction of movement (left or right) of a central moving human stick figure while flanking figures either moved in a direction congruent or incongruent with the central figure. The time to identify the direction of movement of the central figure was slowed in the incongruent condition, indicating that the flanking movements were processed even though they were outside the focus of attention and irrelevant to the task. Some computational models have also supported the view that biological motion perception occurs automatically through bottom-up visual mechanisms (Giese and Poggio, 2002).

Such a view would suggest that the detection of the threatening actions of people and objects should be rapid and efficient, which it often is under optimal viewing conditions. Yet this appears not to be always the case, as indicated by the performance of people watching complex moving images under challenging naturalistic conditions, such as an unmanned air vehicle operators watching for threat-related activity in video imagery. One possibility is that while simple movements and actions may be largely processed automatically in a bottom-up manner, biological motion may be influenced by top-down factors under more demanding viewing conditions, such as when images are degraded due to sensor or communication channel noise, partially obscured by other movements, when threats only occur rarely, and when other factors are present that place demands on operator attention. When viewed in ideal conditions, many movements and actions may be processed with little or no effort. However, under non-optimal or degraded viewing conditions, attention may be required to resolve ambiguity or enhance perceptual processing so that threats are detected.

### Effects of divided attention

Nakayama and Joseph (1998) suggested that many perceptual processes—signal detection, pattern perception, object recognition, etc.—require attentional resources (Norman and Bobrow, 1975), but typically only to a small degree. Consequently, in order to demonstrate that a perceptual process is attention sensitive, the observer's attentional resources may need to be depleted to a large extent by a secondary task. Thornton et al. (2002) used this strategy in a dual-task study in which participants had to discriminate the direction of movement of point-light displays of human walkers while simultaneously performing a highly demanding secondary task—detecting changes in the orientation of four rectangles that surrounded the walkers. The secondary task was presented either with the moving walkers or with noise dots consisting of scrambled motion. The dual-task performance decrement was significantly greater for the walkers than for the noise stimuli, suggesting that determining the direction of movement of human walkers requires a global spatial integration process that is attentionally demanding. Moreover, increasing the interval between successive frames of the moving stimuli—thus making integration of motion information over time more challenging—also increased interference from the secondary task. Both sets of findings suggest that perception of biological motion requires attentional resources. In another study from the same group, accuracy in determining the orientation of point-light actions was found to be inversely correlated with the amount of interference participants exhibited on the Stroop color-word task, a well-known measure of the ability to control attention (Chandrashekharan et al., 2010).

The results of these behavioral studies indicate that attention plays a role in the perception of biological motion, such as determining the direction of movement of human walkers. But threat detection involves more than identifying movements: the *intent* behind the movements, or *action understanding*, must also be identified. Not all movements constitute a threat, only those associated with specific intentional actions aimed at other individuals. Biological motion-based threats could involve movements made by another person, actions performed on an object, or some combination. If attention is necessary to perceive biological motion, especially under less than optimal conditions, is it also required to understand these actions?

### Effects of sustained attention

**Figure 2:**
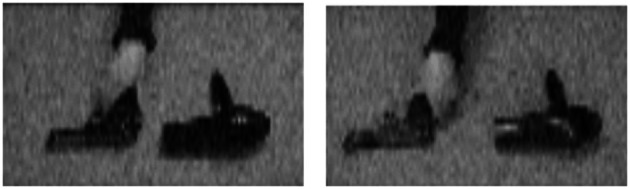
**Examples of still frames from videos depicting grasping actions with a gun or hairdryer.** From Parasuraman et al. (2009).

Parasuraman et al. (2009) examined the issue of the role of sustained attention in action understanding. Participants viewed videos of a person's hand reaching to grasp either a gun or a similarly shaped object (a hairdryer) (Figure 2). The actor (whose face was not shown) grasped the gun or hairdryer in a manner compatible with using either object (utilization intent) or in such a way that it could not be used but only moved from one location to another (transport intent). The object could appear either in the left or right visual field and could point either left or right. Participants were asked to detect a particular target intentional action or threat that occurred infrequently—grasping the gun to use it to fire in a specific direction. All other movements were classified as non-targets or non-threatening events. Participants performed the task over a 22 min period under two conditions, with very low image degradation, so that movements were clearly perceptible, or with high image degradation that made the detection of intent more difficult.

**Figure 3:**
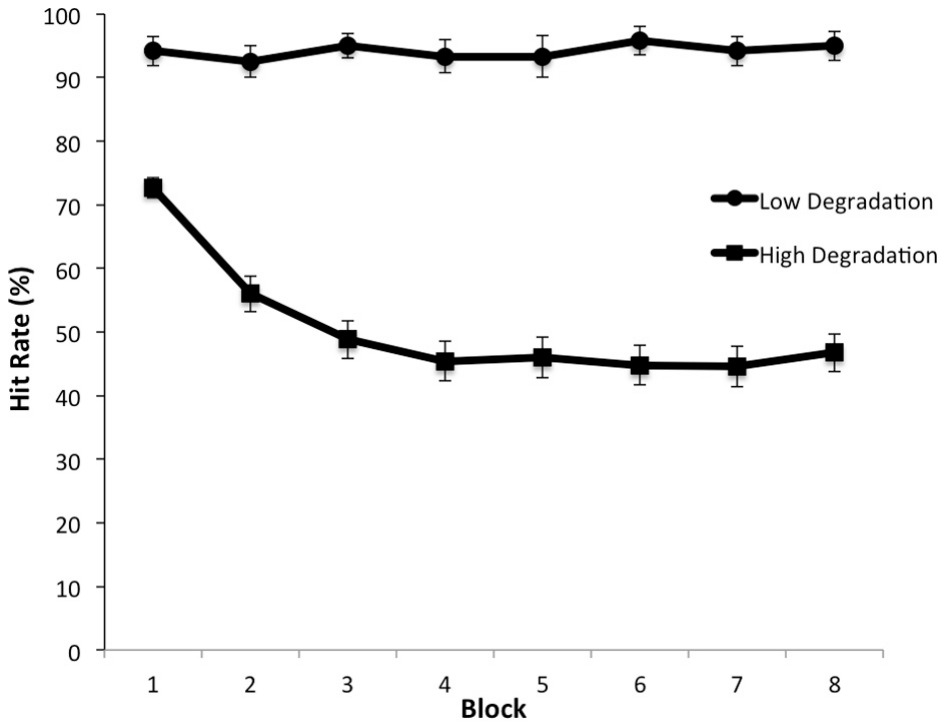
**Detection rate of threatening actions as a function of time on task (blocks) under low and high visual degradation conditions.** From Parasuraman et al. (2009).

When participants viewed videos that were minimally degraded, they were highly accurate in threat detection and showed no decline in performance over time on task. Thus, in this condition, performance was insensitive to any waning of attention over the 22-min duration of the task. However, when the images were degraded, there was a significant decline in hit rate as a function of time—that is, participants exhibited a vigilance decrement (Figure 3). This finding is consistent with other findings that vigilance decrement is increased for targets that are difficult to discriminate (Warm et al., 2008). Furthermore, analysis of the distribution of false alarms—incorrect threat present responses made to non-targets—showed that the requirement to sustain attention over a long period impaired participants' understanding of action intent: false alarms were more frequent for events associated with the wrong intention (e.g., grasping the gun to transport it instead of grasping the gun in order to fire it) than for other non-target events (e.g., using the hairdryer). Thus, the vigilance decrement could not be attributed to participants letting their minds wander (Robertson et al., 1997), as this would predict a random distribution of false alarms over non-target types. Rather, threat detection required effortful allocation of attentional resources, which became depleted over time (Warm et al., 2008).

These findings are consistent with the view that attending to the meaning of an observed action, such as the intention behind the action, is demanding if the stimuli are difficult to discriminate. The role of attention in action recognition is therefore not restricted solely to detecting or discrimination of a specific human action. Instead, if the movements and actions of people and objects occur under degraded viewing conditions, the decoding of inferences based on observed actions is also attentionally demanding. Attention is known to modulate neural activity in brain networks controlling different perceptual processes (Posner and Petersen, 1990; Petersen and Posner, 2012). Activity in brain regions responsible for biological motion perception should therefore also be affected by allocation or withdrawal of attention. We turn to such evidence next.

## Neuroimaging studies

### The action understanding network

**Figure 4:**
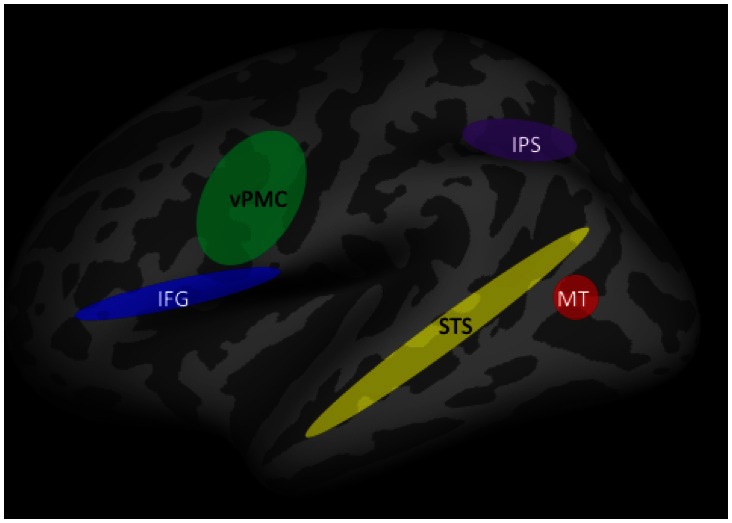
**Brain areas involved in action understanding.** IFG, inferior frontal gyrus; IPS, intraparietal sulcus; MT, middle temporal gyrus; STS, superior temporal sulcus; vPMC, ventral premotor cortex.

Neuroimaging studies using fMRI have revealed that a number of cortical regions within the dorsal and ventral visual processing pathways are associated with biological motion perception and action understanding. Figure 4 shows the major components of the associated cortical networks. It should be noted that while the specific functions that each of these cortical areas mediate have been identified, the coordination and relative timing of neural activity between cortical areas is a continuing topic of research.

Initial encoding of biological motion occurs in regions of the posterior inferior temporal sulcus, in particular the medial temporal area (MT) (Grossman and Blake, 2002; Thompson et al., 2005). It is thought that the processing of features that compose an action may begin in this area, but that action recognition requires integration across features that is carried out in higher-order visual areas, in particular the superior temporal sulcus (STS). The STS is viewed as a critical brain structure for the recognition of human actions (Grossman and Blake, 2002). This was first demonstrated in single-unit recording studies in monkeys (Perrett et al., 1985) and subsequently confirmed in fMRI studies in humans (Grossman and Blake, 2002; Puce and Perrett, 2003). More importantly, the necessity of STS for action understanding was established in a large-sample study of stroke patients with unilateral lesions of STS and inferior frontal cortex but not of other brain regions (Saygin, 2007).

Additional evidence that the STS is important for the formation of action-specific representations comes from studies using the fMRI adaptation technique, in which neural responses to repeated stimuli that belong to the same category are compared to those to novel stimuli (Grill-Spector et al., 2006). Using this method, Grossman et al. (2010) showed that adaptation to repeated actions in STS was independent of the angle from which the actions were viewed, indicating that the STS is associated with the formation of higher-order representations of actions.

Additional processes must supplement the encoding of biological motion and the representation of actions for action *understanding* to occur. These include information about objects being used by another person [e.g., such as a gun or hairdryer as in the previously-described study by Parasuraman et al. (2009)]. Contextual information about the setting, or prior knowledge, are other factors that will influence understanding the meaning of another person's actions and of inferring their intent, including the possibility of threat. Brain regions outside the primary biological motion/action representation regions of the STS appear to be associated with such intention understanding. They include the inferior parietal and inferior frontal cortex. For example, in a study examining neural activity associated with grasping movements, Hamilton and Grafton (2006) showed that activation of the inferior parietal lobule (IPL) and the anterior intra-parietal sulcus (aIPS) was associated with processing the goal of the observed grasping action, rather than the movement kinematics of the action. In addition, the ventral premotor cortex (vPMC) and the inferior frontal gyrus (IFG) also appear to code the intention behind specific actions of others (de Lange et al., 2008; for a review, see Grafton and Tipper, 2012).

While there is good evidence that the brain regions shown in Figure 4 are involved in the encoding, representation, and understanding of the actions of others, how these regions interact together, their relative timing of activation, and the effects of attention, are all not fully understood and are current topics of research. One approach to examining the coordination and relative timing issues is to supplement fMRI with electrophysiological methods such as EEG and MEG that have higher temporal resolution than fMRI. This method was used by Ortigue et al. (2009), who examined fMRI and high-density ERPs during performance of a version of the gun/hairdryer task described previously in the study by Parasuraman et al. (2009). Participants were instructed to attend to a series of 3 s-video-clips displaying a hand using or moving either a gun or hairdryer. They were required to respond rapidly (within 1 s) at the end of the last clip to indicate whether the action was consistent with an intention to use (e.g., fire the gun) or transport the object (e.g., move the hairdryer). The fMRI adaptation technique was also used, so that successive trials either repeated a hand-object interaction that reflected the same intention (e.g., use, use) or a different intention (e.g., use, transport). ERPs were recorded using the same event sequence in a separate session. Ortigue et al. (2009) found that compared to when the intentional action was repeated, novel intentions were associated with greater activation in the STS, IPS, and IFG, the main components of the action understanding network shown in Figure 4. The network for understanding intentions extends beyond earlier visual processing areas involved in feature detection (e.g., object shape and size discrimination). In addition, ERP analysis showed that repeated and novel intentions differed in both early activity (~120 ms) that was localized to the STS and IPS and later activity (~350 ms) that was maximal in the IPS and IFG. These findings suggest that understanding the intent behind the movement and actions of another person, including determination of a threatening intent such as firing a gun, involves a distributed network of neocortical regions. The spatiotemporal dynamics of activation in this network can be specified to a degree. However, is it the case that attention has an influence on components of the network? We turn to this issue next.

### Effects of attention

There are several ways that attention has been manipulated in neuroimaging studies to examine modulation of stimulus-processing cortical areas and the sources of such modulation (Posner and Petersen, 1990; Petersen and Posner, 2012). Previously we described two methods of increasing the attentional demands of biological motion perception, as suggested by Nakayama and Joseph (1998)—requiring participants to perform a challenging secondary task, or asking them to maintain attention to rarely occurring target stimuli in degraded visual images over a long period of time. Both of these attentional challenges are likely to occur in naturalistic threat detection environments. Another method, related to the dual-task technique, is to present other moving stimuli that do not need to be responded to but which compete for the participant's attention because they overlap with the movements that the participant has to process (O'Craven et al., 1999). The use of overlapping stimuli that appear in the same location also allows one to distinguish effects of attention on higher-order representations of biological motion and action from effects of spatial attention, which strongly modulates activity in widespread brain regions (Corbetta and Shulman, 2002).

Safford et al. (2010) used this method to examine whether attention modulated neural activity (using fMRI and ERPs) in the action understanding network when competing, non-biological motion was simultaneously present. Participants viewed videos of human point light motion (e.g., a person doing jumping jacks) that were superimposed on videos of tool motion. Participants were required to perform a 1-back task on either the biological motion or the tool motion, that is, to detect whenever one type of motion was repeated. Thus the task required participants to pay attention to one category of movement. fMRI revealed that activation of the STS was higher when participants attended to biological motion and was strongly suppressed when participants attended to the tool motion, even when biological motion was present but not task-relevant. The data suggested that attention acts on actions at the level of object-based representations, because the only way to select the human actions when they spatially overlapped the tool motion was by using the specific combination of form and motion that define that action. Safford et al. (2010) also recorded ERPs in the same participants and to the same stimuli in a separate session. Source localization analyses revealed that bilateral parietal and right lateral temporal cortices showed early activity at about 200 ms for both biological and tool motion. However, at about 450 ms, greater neural activity in the right STS was observed for biological motion. Moreover, this later neural response to biological motion was strongly modulated by attention. The combined use of fMRI and EEG thus revealed the spatiotemporal characteristics of biological motion perception in the human brain.

A recent study by Hars et al. (2011) provided corroborative neuroimaging evidence for the modulating effects of attention on neural activity in brain regions subserving biological motion perception and action understanding. They had participants who were trained gymnasts watch either naturalistic videos of an expert perform acrobatic gymnastic movements or relatively impoverished point-light displays of the same movements, recorded with a motion capture system and from the same expert. EEG was recorded from 64 scalp sites and analyzed in three frequency bands (4–8, 8–10, and 10–13 Hz). Functional connectivity for the supplementary motor area in the 4–8 and 8–10 Hz frequency bands was greater during the less familiar and more attentional demanding point-light display than for the videos. The authors concluded that experts at understanding particular actions nevertheless require attention to understand those actions when they occur under unfamiliar viewing conditions, as with the point-light displays.

## Brain stimulation studies

The fMRI and ERP studies we have described have identified the key brain regions associated with biological motion perception and action understanding and, to a degree, the temporal dynamics of interactions between different parts of this network. We have also shown that attention modulates neural activity in key cortical regions, such as the STS. The source of such attentional modulation is the frontoparietal attention control network (Posner and Petersen, 1990; Petersen and Posner, 2012). This suggests that in cases where action understanding and threat detection is challenging and prone to error, such as those described previously—visually degraded images, obscured or overlapping movements, secondary tasks that must be performed, etc.—stimulation of the attention control network might be a possible method to boost performance, consistent with the sense-assess-augment framework described previously.

Skill in threat detection typically develops only after extensive training. For example, intelligence analysts looking at satellite imagery for threats or security officers examining surveillance videos of people for suspects may require many months or years to develop their expertise. At the same time, the number of operational settings that demand skilled surveillance operators is increasing day by day. Hence, validated augmentation methods that can accelerate learning and enhance performance in threat detection will meet a critical need.

There are many different techniques that are available for augmenting human perceptual and cognitive performance. These include neuropharmaceuticals or implants to improve alertness or memory (Mackworth, 1965; Warburton and Brown, 1972; Lynch, 2002). Selecting persons based on their genotype (Parasuraman, 2009), or even genetic modification itself, are other somewhat futuristic possibilities. While such methods raise many ethical issues (Farah et al., 2004) and may be questionable to some, potential adversaries may be entirely willing to make use of them without reservation. Developing acceptable ways of using science and technology to augment human performance will become increasingly essential for realizing the benefits that many technologies afford. The current technical maturity of various approaches in this area varies widely, but significant steps to advance and develop early implementations are possible now and over the next decade.

A newly emerging augmentation method is to use non-invasive brain stimulation to modulate neuronal activity. There are many such brain stimulation methods, but the two that have received the greatest empirical scrutiny are Transcranial Magnetic Stimulation (TMS) and transcranial direct current stimulation (tDCS) (Utz et al., 2010; McKinley et al., 2012; Nelson et al., 2013). The latter method uses small DC electric currents (typically 1–2 mA) that are applied to the scalp of the participant either before or during the performance of a cognitive or motor task. Brain stimulation at these current levels is safe for use in healthy subjects for up to about 30 min of stimulation (Bikson et al., 2009). The mechanism by which tDCS influences brain function is not precisely known, but is thought to involve alteration of the electrical environment of cortical neurons, specifically small changes in the resting membrane potential of neurons, so that they fire more readily to input from other neurons (Bikson et al., 2004). A positive (anodal) polarity is typically used to stimulate neuronal function and enhance cognitive or motor performance. Conversely, a negative (cathodal) polarity is used to inhibit neuronal activity.

A number of tDCS studies have shown that it is possible to enhance human performance through the application of low-level DC current to the scalp while participants are engaged in simple perceptual, cognitive, and motor tasks (see Utz et al., 2010, for a review). Recently, Pavlidou et al. (2012) also reported improvement in discrimination of point-light stimuli depicting human and animal motion with tDCS of premotor cortex. However, they also reported that tDCS increased false alarms in their discrimination task, so that it is unclear whether tDCS can reliably enhance perceptual sensitivity (in the signal detection theory sense; Green and Swets, 1966), or whether it just lowers the threshold for detection. If the latter were true, it would not support the potential use of tDCS for augmenting threat detection. If both correct and false reports of threat increase with tDCS, threat detection efficiency would not be increased. Moreover, to evaluate whether tDCS can be an effective augmentation technique for threat detection, it should be examined in threat detection tasks with complex targets and naturalistic scenes. Finally, for tDCS to be a viable augmentation technique, its effects should not be transient but should last for some time, preferably for hours if not days.

**Figure 5:**
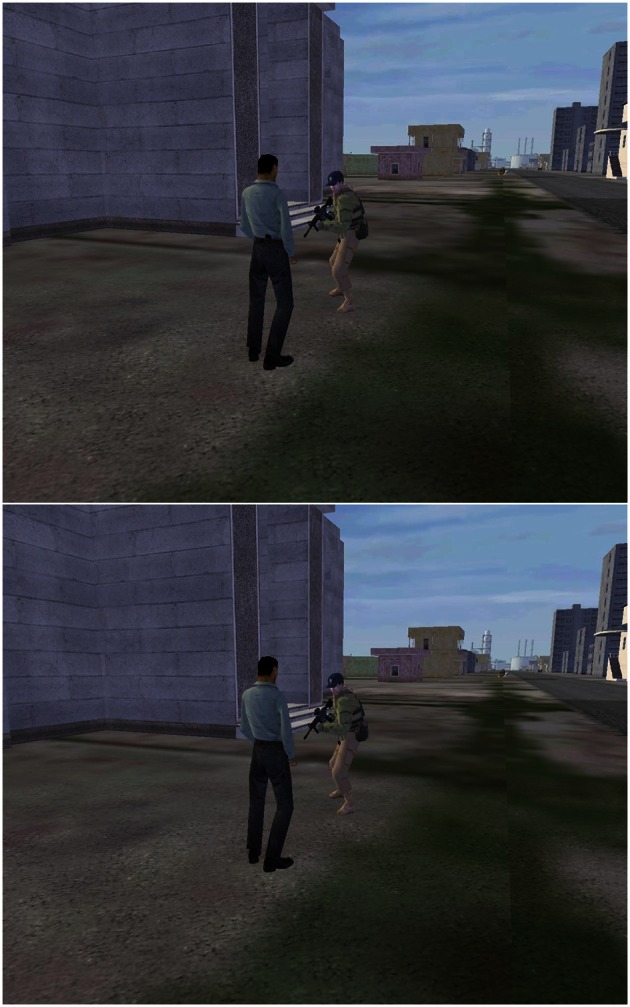
**Examples of images indicating threat (top) and non-threat (bottom) situations.** From Falcone et al. (2012).

A recent study by Falcone et al. (2012) addressed these issues. They examined whether tDCS would improve performance in a complex threat detection task and thereby accelerate learning. Signal detection theory analysis was used to examine effects of brain stimulation on perceptual sensitivity independently of bias. Furthermore, retention of any tDCS benefit on threat detection was assessed by testing participants immediately following and 24 h after brain stimulation. Participants were shown short videos of naturalistic scenes containing movements of soldiers and civilians that were taken from the “DARWARS Ambush” virtual reality software (MacMillan et al., 2005). Half of the scenes included possible threats that participants had to detect, while the other half did not. Examples of threats and non-threats are shown in Figure 5. Participants were only told that they were to determine whether or not there was a threat present in the image, without being provided specific details as to what types of possible threats were present. For example, Figure 5 (top) shows a threat involving a plainly clothed civilian with a concealed weapon behind his back (in his belt). The corresponding non-threat is shown in Figure 5 (bottom). Other examples of threats were a sniper about to fire from a hidden location or a civilian sneaking up behind military personnel. In all cases, non-threats showed the same elements of the scene but without the critical object or movement that constituted the threat. Threat stimuli were subtle enough to be missed on first viewing but could be better identified with training.

During training participants were required to make a button press within 3 s of stimulus onset to indicate whether the scene contained a threat or a non-threat. After each response a short feedback video was presented for all four outcomes: hit, miss, false alarm, or correct rejection. If a threat was present and the participant correctly reported it (a hit), the movie showed the scene progressing without harm and simultaneously a computer-generated voice-over complimented the participant. If a threat was present in the image but the participant missed it, the feedback movie showed the consequence of the failure to detect the threat (e.g., vehicle explosion, friendly casualty, building being destroyed). On a non-threat trial, if the participant responded that a threat was present (false alarm), the voice-over chastised the participant. Finally, if the participant correctly indicated that no threat was present on a non-threat trial (correct rejection), the voice-over praised the participant for correct response. None of these feedback videos provided specific information as to the identity of the threats. Participants were given four training blocks of 60 trials each. Each training block contained 60 trials, approximately half of which contained threats, and lasted 12 min. Test blocks were given before and after training and were similar to training blocks, except that no feedback was given after each response.

Anodal tDCS was applied to the electrode site F10 in the 10–10 EEG system, over the right sphenoid bone. The cathode was placed on the contralateral (left) upper arm. The site of the anode was selected based on previous fMRI results showing that this region of the frontal cortex was the primary locus of neural activity associated with performance of this task (Clark et al., 2012). This brain region is also part of the frontoparietal attention network. Hence, Falcone et al. (2012) reasoned that stimulation of this region with tDCS could serve to provide additional top-down attention control signals to the action understanding network and hence boost threat detection performance. Participants were randomly assigned to either active (2 mA current) or sham stimulation (0.1 mA) from the tDCS unit for a total of 30 min during the first two training blocks, beginning 5 min before the training started.

**Figure 6:**
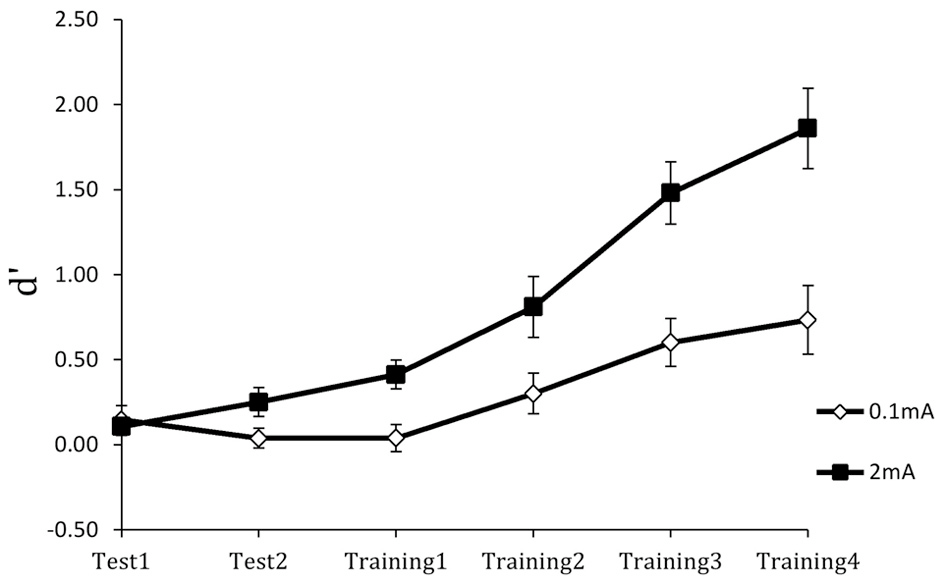
**Perceptual sensitivity (*d*′) of threat detection across test and training blocks for active (2 mA) and sham (0.1 mA) brain stimulation groups.** From Falcone et al. (2012).

**Figure 7:**
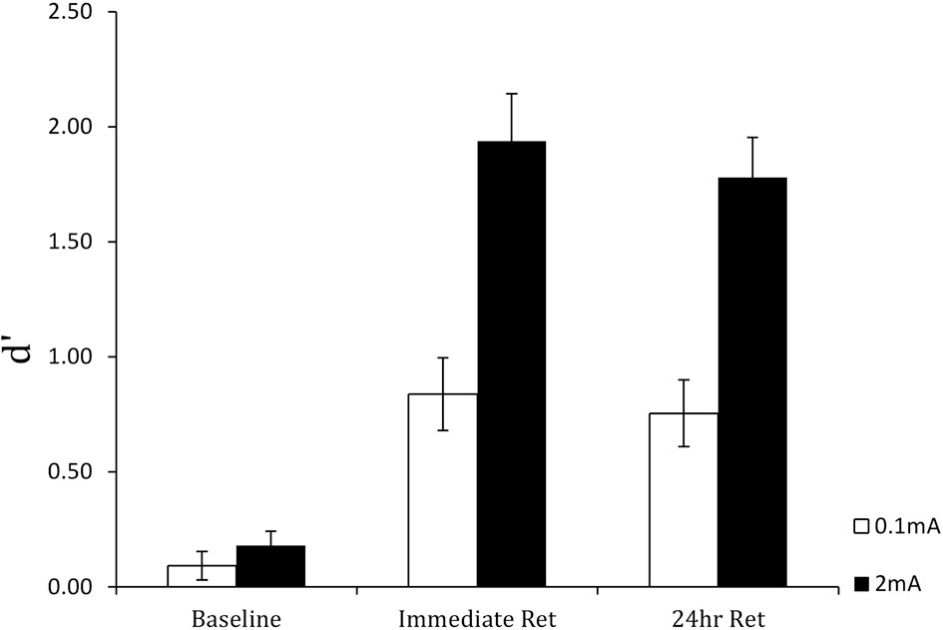
**Perceptual sensitivity (*d*′) of threat detection in the pre-training baseline, immediate post-training retention test, and 24-hour retention test blocks for active (2 mA) and sham (0.1 mA) brain stimulation groups.** From Falcone et al. (2012).

Figure 6 shows the results for the perceptual sensitivity measure *d*′. Compared to the 0.1 mA sham stimulation control, stimulation with 2 mA tDCS increased perceptual sensitivity in detecting targets and accelerated learning. As expected, performance was near chance in both groups at the beginning of training. However, the performance gain with tDCS was extensive: on completion of training, participants in the active stimulation group had more than double the perceptual sensitivity of the control group. Furthermore, there were no group or training effects on the response bias measure β, indicating that tDCS improved the actual efficiency of threat detection. Finally, the performance enhancement was maintained for 24 h, as shown in Figure 7. Following cessation of brain simulation training, threat detection sensitivity remained at a high level (immediate retention). Furthermore, while there was some forgetting when participants returned for testing a day later, 24-h retention remained relatively high. This last finding bodes well for the use of tDCS as a training method with potentially lasting effects, although retention over longer periods of days and months will need to be demonstrated.

## Conclusions

Civilian and military operations in the field of security depend on efficient interaction between technological systems and their human operators. Although the final decision in security-related tasks such as threat detection is typically placed in human hands, machine detection and analysis represent important inputs that are used by human decision makers. Thus, the overall efficiency of the human-machine system depends on the cognitive and affective characteristics of human operators. In this paper we have proposed that improving threat detection in these environments requires a number of steps. First, analysts must sense when less than ideal conditions exist for the human operator in a threat detection task. Second, threat detection performance in that condition must be assessed relative to an expected standard. Third, augmentation methods must be applied if the standard is not met.

Behavioral and neuroimaging studies of sensing and assessment of humans performing threat detection tasks show that attention plays an important role in action identification and understanding. Attention is critically important when operators have to view images that are obscured by other objects or movements, or are visually degraded, when other tasks compete for the operator's attention, or when threats occur infrequently over a prolonged period of surveillance–all features that are characteristic of security-related operations.

Neuroimaging studies reveal that action understanding recruits the superior temporal cortex, intraparietal cortex, and inferior frontal cortex. Within this network, attention modulates activation of the STS and middle temporal gyrus. The dorsal frontoparietal network may provide the source of attention-modulation signals to action representation areas. If sensing and assessment of the human operator reveals attention to be a limiting factor in threat detection, stimulation of the attention network provides a method for augmenting performance. tDCS represents one such augmentation method. tDCS of the frontoparietal network boosts top-down attention control signals that can enhance the detection and identification of threat-related actions.

The cognitive, neuroimaging, and brain stimulation studies we have described provide converging evidence for the critical role of attention in threat detection. As such, these studies are a starting point for a deeper understanding of the neurocognitive mechanisms of threat detection. Although some of the studies we described used naturalistic scenes and videos, additional work needs to be done with even more realistic scenarios and under conditions that better approximate threat detection in real-world security operations.

### Conflict of interest statement

The authors declare that the research was conducted in the absence of any commercial or financial relationships that could be construed as a potential conflict of interest.
